# Creative Minds: San Francisco's Community Arts for Brain Health Initiative

**DOI:** 10.1002/alz70860_106898

**Published:** 2025-12-23

**Authors:** Gloria Annette Aguirre, Magda Kaczmarska, Alexander N Kornhuber, Stefanie D Pina‐Escudero, Olakunle Joel Adewale, Alinda Amuiri, Jake Broder, Catherine Correa Lopera, Tanisha G Hill‐Jarrett, Ashley J. Jackson, Kevin Lieu, Luis E Martinez, Diana Mei, Caroline Prioleau, Veronica B Rojas Carstensen, Mollie Rose, Dvera I Saxton, Charles C. Windon, Victor Valcour, Bruce L. Miller, Serggio Lanata

**Affiliations:** ^1^ Memory and Aging Center, UCSF Weill Institute for Neurosciences, University of California, San Francisco, San Francisco, CA, USA; ^2^ Global Brain Health Institute, San Francisco, CA, USA; ^3^ Global Brain Health Institute, University of California, San Francisco, CA, USA, San Francisco, CA, USA; ^4^ Memory and Aging Center, Weill Institute for Neurosciences, University of California San Francisco, San Francisco, CA, USA; ^5^ University of California, San Francisco, San Francisco, CA, USA

## Abstract

**Background:**

The rising prevalence of age‐related neurocognitive disorders (NCDs) poses significant public health challenges, particularly in diverse urban populations with social and structural barriers to healthcare. Research suggests that social engagement and physical activity are key modifiable risk factors for dementia prevention. Creative Minds (CM), launched in 2020, is San Francisco's first neighborhood‐based arts initiative for brain health. CM is designed to mitigate social isolation and sedentarism in older adults by integrating artistic engagement with artist‐ and physician‐led workshops and educational activities, thereby promoting brain health literacy, socialization, and access to clinical care and research.

**Method:**

CM is a community‐based program that provides culturally tailored, multilingual creative arts workshops in underserved neighborhoods of San Francisco in collaboration with the Global Brain Health Institute (GBHI) and community partners. Facilitated by professional artists**,** neurologists and geriatricians, CM engages participants in activities including visual arts, photography, dance, and storytelling. Educational components include physician‐led discussions on brain health, AD/ADRD risk factors, and discussions on the importance of participating in clinical research. Workshops are delivered in‐person and virtually, ensuring accessibility. Program effectiveness is assessed via pre‐ and post‐workshop surveys evaluating social engagement, health literacy, and willingness to participate in dementia prevention research.

**Result:**

From 2020 to 2023, we conducted 280 CM workshops, reaching 156 older adults across multiple San Francisco neighborhoods. 70 educational sessions were delivered, with an increasing number of arts participants connected to AD/ADRD‐related research opportunities, demonstrated by a sample group of ten visual arts participants established in 2020 that has resulted in nine referrals and eight enrollments in brain health and aging research programs at the Memory and Aging Center, with deep characterizations including biomarkers. Preliminary CM survey data indicate improvements in self‐reported social connectedness, engagement in cognitively stimulating activities, and increased awareness of brain health practices. Additionally, CM's culturally tailored approach fostered greater engagement from monolingual and immigrant communities.

**Conclusion:**

Community‐based arts engagement programs like CM offer a scalable, low‐cost intervention to promote brain health, address social determinants of AD/ADRD risk, and improve healthcare and research access in vulnerable populations. Future directions include expanding CM's reach and integrating additional evaluation measures.

